# Transcriptomic Analyses of Tomato Exhibiting Induced Resistance to *Ralstonia solanacearum* by *Lysobacter enzymogenes* JCK1421

**DOI:** 10.3390/plants14223415

**Published:** 2025-11-07

**Authors:** Jungwook Park, Hyejung Jung, Taeho Jeong, Ae Ran Park, Mohamed Mannaa, Duyoung Lee, Jin-Cheol Kim, Young-Su Seo

**Affiliations:** 1 Department of Integrated Biological Science, Pusan National University, Busan 46241, Republic of Korea; jjuwoogi@korea.kr (J.P.); jhj4059@pusan.ac.kr (H.J.); anqmsl@pusan.ac.kr (T.J.); mannaa@cu.edu.eg (M.M.); dlendud2164@pusan.ac.kr (D.L.); 2 Biotechnology Research Division, National Institute of Fisheries Science, Busan 46083, Republic of Korea; 3 JAN153 Biotech Incorporated, Gwangju 61186, Republic of Korea; arpark9@naver.com; 4 Division of Applied Bioscience and Biotechnology, Chonnam National University, Gwangju 61186, Republic of Korea; 5 Department of Plant Pathology, Faculty of Agriculture, Cairo University, Giza 12613, Egypt; 6 Institute of System Biology, Pusan National University, Busan 46241, Republic of Korea

**Keywords:** comparative transcriptome analysis, crop protection, induced resistance, plant–pathogen interactions

## Abstract

*Lysobacter enzymogenes* is well known for producing extracellular enzymes and bioactive molecules that suppress a wide range of plant pathogens, including fungi such as *Rhizoctonia* and *Fusarium* spp. and oomycetes such as *Phytophthora infestans*. It also exhibits antagonistic effects against Gram-negative bacteria through the type IV secretion system. Interestingly, *L. enzymogenes* JCK1421, isolated from the rhizosphere of pine forests, showed neither antifungal nor antibacterial activity, in contrast to other *L. enzymogenes* strains. However, foliar application of JCK1421 significantly reduced disease symptoms in tomato seedlings challenged with *Ralstonia solanacearum*. To elucidate the underlying defense mechanisms, comparative transcriptome analysis integrated with network and pathway enrichment approaches was performed. Comparative transcriptome and network analyses identified signaling modules activated by JCK1421 in pathogen-free plants and further enhanced upon *R. solanacearum* challenge. In challenged plants, JCK1421 treatment strongly induced resistance-related genes, including those encoding Ca^2+^-dependent proteins and ion channels, hormone biosynthesis components, and mitogen-activated protein kinase cascades—hallmarks of plant immune responses. These findings demonstrate that JCK1421 provides an effective model for investigating microbe-associated defense activation in plants, highlighting its potential as an eco-friendly agent for sustainable crop protection.

## 1. Introduction

Plant growth-promoting rhizobacteria (PGPR) contribute to plant growth and protection by supplying essential nutrients and enhancing stress tolerance [1,2]. PGPR-mediated effects on plant growth and stress responses involve the modulation of hormone and metabolite levels and the activation of associated signaling pathways [3]. PGPR confers tolerance to abiotic stresses, such as drought and salinity, and to biotic stresses by suppressing diverse plant pathogens. This suppression can occur either through direct inhibition of pathogens or via the induction of systemic resistance, which provides broad-spectrum protection against multiple pathogens [4,5]. Local and systemic defense responses triggered by beneficial microbes are coordinated by a complex signaling network in which the plant hormones salicylic acid (SA), jasmonic acid (JA), and ethylene (ET) play central roles [6]. Increasing evidence indicates that the SA, JA, and ET pathways interact to finely regulate plant defense responses against pathogen infection [7].

*Lysobacter*, a genus within the family Xanthomonadaceae, comprises Gram-negative bacteria that exhibit strong antagonistic activity against diverse plant pathogens [8,9]. Among them, *Lysobacter enzymogenes* has been extensively studied due to its production of extracellular enzymes and bioactive molecules. *L. enzymogenes* is effective against several fungal pathogens, including *Rhizoctonia* and *Fusarium* spp. [10,11], as well as oomycete pathogens such as *Phytophthora infestans* and *Pythium aphanidermatum*. This activity is largely attributed to the secretion of lytic enzymes, such as chitinases, and the production of specialized natural products, such as the heat-stable antifungal factor (HSAF) [12,13]. In addition, cyclic lipodepsipeptides such as WAP-8294As act as antagonistic effectors against Gram-positive bacteria [14]. Although *L. enzymogenes* does not produce antibiotic metabolites effective against Gram-negative bacteria, it can efficiently eliminate bacterial competitors via a contact-dependent mechanism mediated by the type IV secretion system (T4SS) [15,16]. The T4SS effector protein LtaE is translocated into *Pseudomonas* species, where it interacts with the transcriptional repressor PhlF and the transcriptional regulator LuxR1, thereby disrupting host defense systems [16].

In addition to seed treatment, foliar spraying of PGPR can be applied at critical growth stages to enhance nutrient uptake and disease resistance by directly targeting leaves [17]. In this study, foliar application of *L. enzymogenes* JCK1421 to tomato seedlings significantly reduced disease symptoms caused by *Ralstonia solanacearum* compared with untreated controls. Notably, unlike other *L. enzymogenes* strains, JCK1421 showed no direct antagonistic effects against plant-pathogenic fungi such as *Fusarium* spp. or Gram-negative bacteria, including *R. solanacearum* and *Burkholderia glumae*, in competition assays, whereas direct antagonism has been reported for other *L. enzymogenes* strains [10,11,12,13].

This study aimed to elucidate the JCK1421-mediated defense mechanisms in tomato against *R. solanacearum* using genome-wide transcriptome analysis integrated with network construction and pathway enrichment. This approach provides new insights into the induction of plant defense responses by *L. enzymogenes* JCK1421, a bacterial isolate that exhibited no detectable in vitro antimicrobial activity under the tested conditions.

## 2. Results

### 2.1. Enhanced Disease Resistance to R. solanacearum in Tomato Seedlings by L. enzymogenes JCK1421

**Figure 1:**
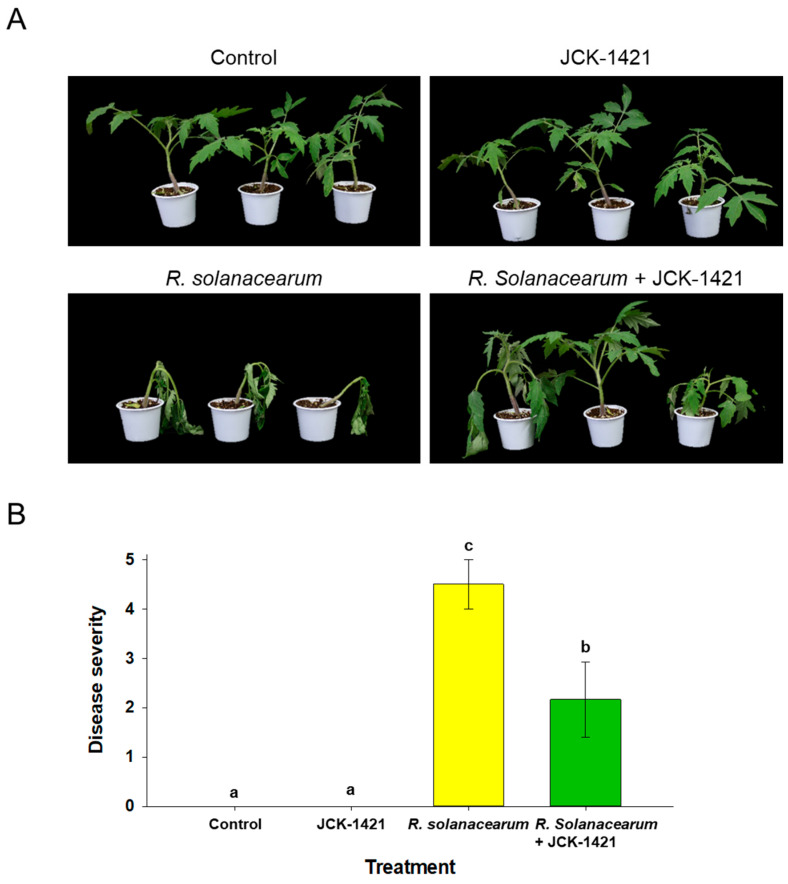
**Effect of *L. enzymogenes* JCK1421 foliar spray on resistance to *Ralstonia solanacearum* in tomato seedlings.** (**A**) Representative phenotype at 14 days post-inoculation. (**B**) Disease severity (0–5 index) scored at 14 days. Bars show mean ± SD; different letters indicate significant differences among treatments based on LSD test, *p* < 0.05.

In the seedling assays, only *L. enzymogenes* JCK1421-treated seedlings remained healthy, comparable to the uninoculated control. By 14 days post-inoculation, *R. solanacearum* caused severe wilting and collapse in infected seedlings. In contrast, seedlings treated with JCK1421 one week before pathogen challenge with *R. solanacearum* displayed enhanced disease resistance, exhibiting markedly reduced wilting at 14 days post-inoculation (Figure 1A). Disease severity scores (0–5 scale) corroborated these observations and differed significantly among treatments (*p* < 0.05; Figure 1B). No significant difference in growth or vigor was observed between JCK1421-treated and untreated seedlings, indicating that the treatment itself did not affect plant growth under non-stress conditions. *R. solanacearum* alone averaged 4.50, and the co-treatment (JCK1421 + *R. solanacearum*) averaged 2.16.

### 2.2. In Vitro Interactions Between L. enzymogenes JCK1421 and Plant Pathogens

A dual-culture assay on YMA medium was performed to evaluate the antifungal activity of *L. enzymogenes* JCK1421 against *Fusarium oxysporum*, *F. fujikuroi*, *Botrytis cinerea*, and *Colletotrichum scovillei*. In parallel, a spot-lawn assay was employed to test the antagonistic activity of JCK1421 against plant-pathogenic bacteria, including *Burkholderia glumae*, *Pseudomonas syringae* pv. *tomato* DC3000, *Pectobacterium carotovorum*, and *R. solanacearum*. In all assays, JCK1421 failed to inhibit the growth of either pathogenic fungi or bacteria (Figure S1).

### 2.3. Overview of RNA-Seq Data from Tomato Plants

RNA-seq libraries were generated from tomato seedlings under four experimental conditions: untreated control (C), pathogen-inoculated (P), JCK1421-treated (T), and co-inoculated with JCK1421 and pathogen (PT). Each condition included three biological replicates, yielding a total of 12 libraries (24 sequencing datasets). Paired-end 2 × 151 bp sequencing on the Illumina NovaSeq platform produced 20.79–21.10 million raw reads per library end, corresponding to ~75.96 Gbp across all datasets (Table S1). GC content ranged narrowly from 41.9% to 43.4%, indicating minimal compositional bias among samples. Base-calling quality was consistently high, with Q30 bases averaging 91.22 ± 0.69%.

Following adapter removal, short reads (<100 bp) and low-quality reads with ≥50% bases below Phred 28 were discarded. Trimming retained 94.31 ± 1.21% of bases (range: 91.92–95.54%), yielding an average of 20.15 ± 0.26 million high-quality reads per end (Table S2). Trimmed reads were aligned to the *Solanum lycopersicum* genome SL4.0 with ITAG4.0 annotation (release 2019-09-06), as summarized in Table 1. Alignment performance averaged 96.93 ± 1.07% (93.72–97.96%) relative to trimmed reads, corresponding to 19.53 ± 0.37 million mapped reads per end (Table S2). These results confirm the generation of high-quality RNA-seq datasets suitable for downstream co-expression and network analyses.

### 2.4. Construction of a Co-Expression Network and Module Detection

A weighted gene co-expression network analysis (WGCNA) was performed to characterize treatment-dependent transcriptional programs. From the initial 34,075 annotated *loci*, 24,367 genes with detectable expression across the 12 libraries were retained for network inference (Table S3). Gene expression values were normalized to RPKM and transformed to log_2_(RPKM + 1) to stabilize variance before correlation and adjacency calculations.

**Figure 2:**
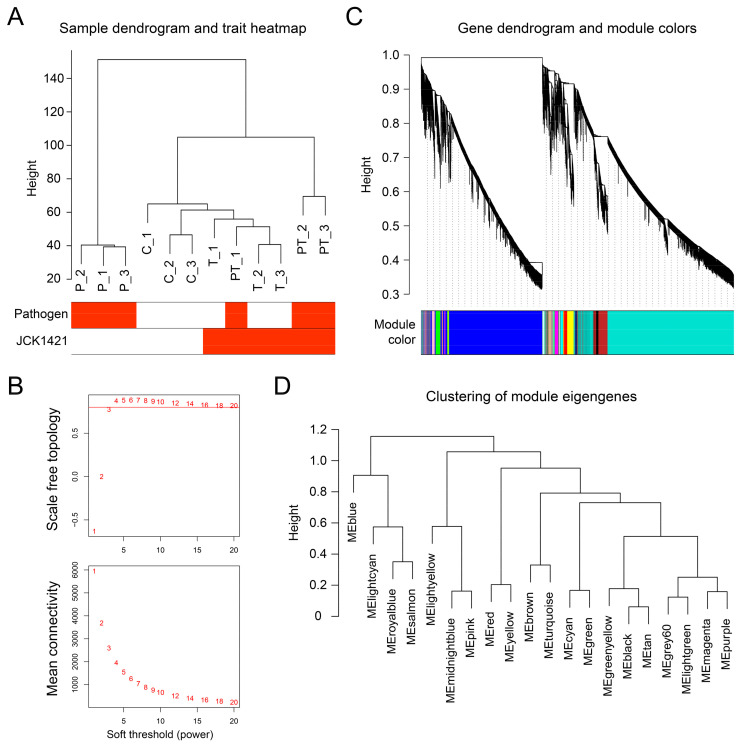
**Exploration of co-expressed gene modules through WGCNA.** (**A**) Sample dendrogram based on transcriptome profiles from four tomato treatment groups: untreated control (C), pathogen-inoculated (P), JCK1421-treated (T), and co-treatment with pathogen and JCK1421 (PT). The heatmap below the dendrogram indicates the treatment status of each sample for pathogen and JCK1421. (**B**) Determination of the optimal soft-thresholding power for scale-free topology in network construction. The upper plot shows the scale-free topology fit index (*y*-axis) across soft-thresholding powers (*x*-axis). A threshold of 0.8 was used as the criterion for selecting the power value (red line). The lower plot shows mean connectivity, which decreases as power increases. (**C**) Gene dendrogram generated by hierarchical clustering of topological overlap matrix (TOM) dissimilarity. Colored bars beneath the dendrogram represent distinct gene co-expression modules. (**D**) Dendrogram of module eigengenes summarizing the correlation relationships among identified modules. Modules with similar expression profiles are clustered together based on eigengene dissimilarity.

Unsupervised hierarchical clustering of the full expression matrix recapitulated the experimental design (Figure 2A). Replicates of the pathogen treatment (P) formed a distinct clade, while JCK1421-treated samples (T) clustered closely with the control (C). Co-inoculated samples (PT) formed a separate cluster, distinct from both C/T and P, reflecting unique transcriptional reprogramming under combined treatment. No outlier libraries were detected, and all were retained for network construction.

Scale-free topology analysis identified a soft-thresholding power (*β*) of 4 as the lowest value at which the signed R^2^ exceeded 0.80 while maintaining adequate connectivity (Figure 2B; Table S4). Using this threshold, dynamic tree cutting on the topological overlap matrix (TOM) resolved 20 distinct co-expression modules, each assigned a unique color label (Figure 2C). Module sizes varied substantially, ranging from 68 genes in the royal blue module to 11,055 genes in the turquoise module (Table S5). Clustering of module eigengenes (MEs) further revealed inter-modular relationships and higher-order co-expression patterns (Figure 2D).

### 2.5. Co-Expression Modules Correlated with Experimental Traits

**Figure 3:**
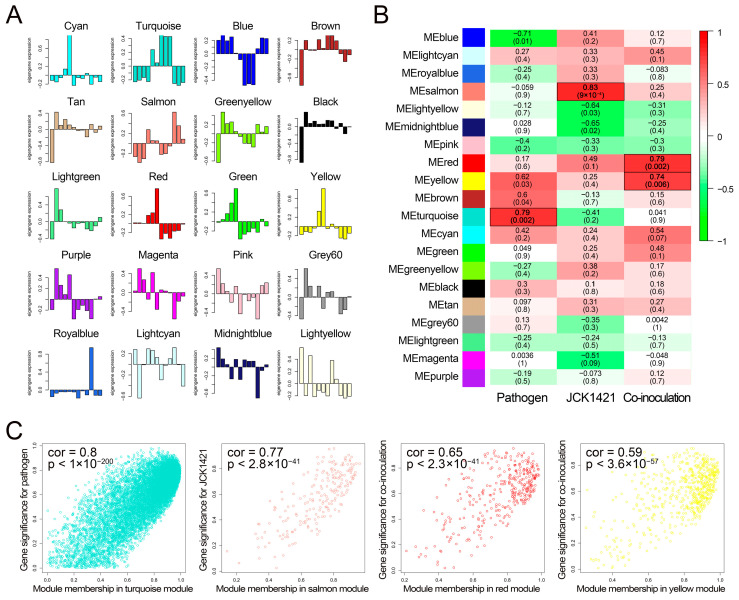
**Identification of WGCNA modules associated with pathogen infection and JCK1421 treatment.** (**A**) Bar plots showing eigengene expression profiles for each co-expression module across 12 tomato transcriptome samples. Each plot represents eigengene values (first principal component) of a module, reflecting the overall expression pattern of genes. (**B**) Heatmap displaying the Pearson correlation between module eigengenes and experimental treatments (pathogen, JCK1421, and co-inoculation). The color scale ranges from green (negative correlation) to red (positive correlation), with deeper colors indicating stronger correlation values. The top number in each cell indicates the correlation coefficient, and the value in parentheses indicates the corresponding *p*-value. (**C**) Scatter plots showing the relationship between module membership (*x*-axis) and gene significance (*y*-axis) for four selected modules: turquoise, salmon, red, and yellow. Each dot represents a gene. The overall correlation coefficient and *p*-value are shown in the upper left corner of each panel.

To investigate the association between gene co-expression modules and experimental traits, ME profiles were first examined across all 20 detected modules (Figure 3A). Correlation analyses between MEs and experimental traits identified four treatment-associated modules at *p* < 0.01 (Figure 3B): the turquoise module (11,055 genes) showed the strongest positive correlation with pathogen inoculation (cor = 0.79, *p* = 0.002); the salmon module (204 genes) correlated with JCK1421 treatment (cor = 0.83, *p* = 9.0 × 10^−4^); and, for co-inoculation, the red (333 genes; cor = 0.79, *p* = 0.002) and yellow (596 genes; cor = 0.74, *p* = 0.006) modules were significantly associated.

The robustness of these associations was supported by strong relationships between gene significance and module membership (Figure 3C). Genes within the turquoise module showed a strong linear relationship between pathogen-related significance and module membership (cor = 0.80, *p* < 1 × 10^−200^), suggesting that highly connected genes are responsive to pathogen inoculation. Similarly, salmon module genes displayed a high correlation between JCK1421-related significance and module membership (cor = 0.77, *p* = 2.8 × 10^−41^). In the red (cor = 0.65, *p* = 2.3 × 10^−41^) and yellow (cor = 0.59, *p* = 3.6 × 10^−57^) modules, gene significance for co-inoculation was closely aligned with module membership. These results suggest that the red/yellow and salmon modules play crucial roles in the interactions between JCK1421 and tomato, contributing to defense against pathogen infection.

### 2.6. Biological Pathways Enriched in Target Modules

KEGG pathway enrichment was performed on the four treatment-associated modules. The yellow module (co-inoculation) was enriched for 17 pathways (Table 2), prominently including plant–pathogen interaction (map04626; *p* = 6.56 × 10^−8^), mitogen-activated protein kinase (MAPK) signaling pathway—plant (map04016; *p* = 4.41 × 10^−6^), and plant hormone signal transduction (map04075; *p* = 3.0 × 10^−4^). Additional enrichments included glycerophospholipid metabolism (map00564), α-linolenic acid metabolism (map00592), and ABC transporters (map02010), suggesting enhanced lipid-derived signaling and defense metabolite mobilization.

The red module (co-inoculation) showed eight enriched pathways (Table 2), led by plant hormone signal transduction (map04075; *p* = 1.0 × 10^−4^), with additional support for plant–pathogen interaction (map04626; *p* = 0.0017) and NOD-like receptor signaling (map04621; *p* = 0.0342). Together, the yellow and red modules indicate multilayered activation of hormone-coupled and pathogen-responsive signaling during co-inoculation with the pathogen and JCK1421.

The salmon module (JCK1421 only) exhibited nine enriched pathways (Figure S2). Among them, plant hormone signal transduction (map04075; *p* = 0.00025) emerged as the most significant pathway, implicating the activation of hormone-driven cascades during systemic defense priming by JCK1421 treatment. Additional enrichments (e.g., map00460, cyanoamino acid metabolism; map01120, microbial metabolism in diverse environments; map00720, carbon fixation) suggest metabolic shifts associated with the primed physiological state.

In contrast, the turquoise module (pathogen only) was enriched for processes related to protein homeostasis and trafficking (Figure S3), including protein processing in the ER (map04141; *p* = 1.14 × 10^−4^), proteasome (map03050; *p* = 9.78 × 10^−5^), and ubiquitin-mediated proteolysis (map04120; *p* = 0.0020), as well as endocytosis (map04144), SNARE-mediated vesicular transport (map04130), and phagosome (map04145). These results are consistent with roles in intracellular protein quality control and reorganization during biotic stress caused by pathogen infection. Enrichment of oxidative phosphorylation (map00190), the TCA cycle (map00020), and glycerophospholipid metabolism (map00564) further suggests elevated energetic and membrane remodeling demands under pathogen challenge.

### 2.7. JCK1421-Associated Defense Mechanisms

**Figure 4:**
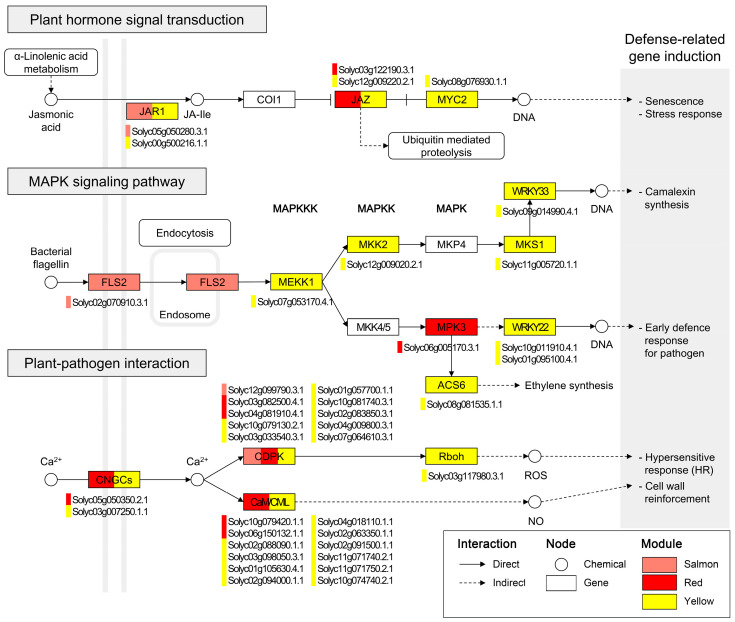
**Key signaling pathways enriched in JCK1421-associated genes.** KEGG pathway enrichment results for genes in the salmon, red, and yellow modules associated with JCK1421 treatment. Three major pathways were identified: plant hormone signal transduction (map04075), MAPK signaling pathway—plant (map04016), and plant–pathogen interaction (map04626). Rectangular nodes represent genes, circular nodes indicate chemical compounds, and rounded rectangles denote pathway categories. Colored bars within gene nodes indicate module membership: red (red module), salmon (salmon module), and yellow (yellow module). Genes assigned to multiple modules contain multiple-colored bars. Solid and dashed lines represent direct and indirect interactions, respectively.

To resolve defense mechanisms associated with *L. enzymogenes* JCK1421 treatment, genes from the salmon, red, and yellow modules were mapped onto molecular networks. Among the enriched results, genes from these modules demonstrated complete representation in the plant hormone signal transduction (map04075), plant–pathogen interaction (map04626), and MAPK signaling (map04016) pathways (Figure 4).

Within the plant hormone signal transduction pathway, genes from three modules were linked to core components of the jasmonic acid (JA)-dependent signaling cascade: jasmonate resistance 1 (JAR1) (Solyc05g050280.3.1; Solyc00g500216.1.1), jasmonate ZIM-domain (JAZ) family members (Solyc03g122190.3.1; Solyc12g009220.2.1), and MYC2 (Solyc08g076930.1.1), implicating JA-dependent transcriptional reprogramming in JCK1421-induced defense. For the MAPK signaling pathway, the salmon module mapped to FLS2 (Solyc02g070910.3.1), a receptor for bacterial flagellin, while the red and yellow modules contained kinase tiers MEKK1 (Solyc07g053170.4.1), MKK2 (Solyc12g009020.2.1), and MPK3 (Solyc06g005170.3.1), with downstream transcription factors WRKY22 (Solyc10g011910.4.1; Solyc01g095100.4.1) and WRKY33 (Solyc09g014990.4.1), outlining a canonical cascade.

Furthermore, in the plant–pathogen interaction pathway, genes from the three modules were positioned at several nodes of calcium signal transduction, including cyclic nucleotide-gated ion channels (CNGCs) and calcium-dependent protein kinases (CDPKs). Calmodulin (CaM) and calmodulin-like (CML) proteins—key mediators of calcium signaling involved in processes such as cell wall reinforcement and the hypersensitive response (HR)—were also represented. These findings indicate that *L. enzymogenes* JCK1421-associated genes occupy central regulatory nodes spanning early pathogen perception, intracellular signaling cascades, and downstream defense responses.

### 2.8. Hub Genes Identified in Target Modules

**Figure 5:**
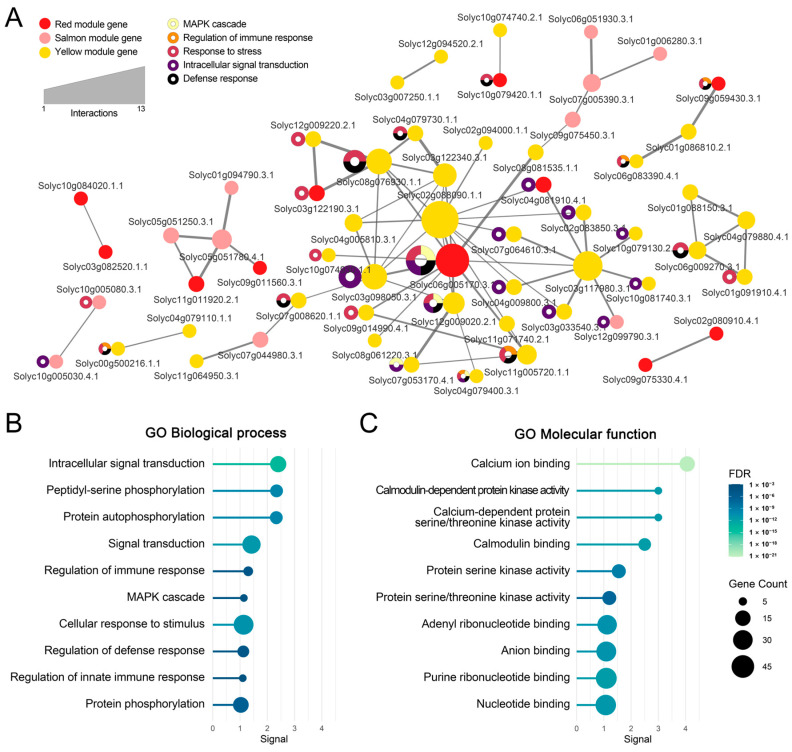
**Functional network analysis of genes within JCK1421-associated modules.** (**A**) Protein–protein interaction (PPI) network of genes in the salmon, red, and yellow modules associated with JCK1421 treatment, constructed using STRING reference data. Each circular node represents a gene, colored by module assignment. Node size reflects the number of interactions (degree), and edge thickness indicates interaction confidence. Donut charts on selected nodes highlight enriched Gene Ontology (GO) biological processes, including MAPK cascade (light yellow), regulation of immune response (orange), response to stress (dark pink), intracellular signal transduction (purple), and defense response (black). (**B**) GO enrichment results for biological processes using genes from the PPI network in (**A**). The *x*-axis indicates the enrichment score (signal); dot size reflects gene count per term, and color represents the false discovery rate (FDR). (**C**) GO enrichment results for molecular function using the same gene set. Dot size and color correspond to gene count and FDR, respectively, as indicated in the legend.

A protein–protein interaction (PPI) network based on the STRING database was constructed from the 106 genes in the enriched pathways (Figure 5A). The most interconnected subnetwork comprised 59 nodes and 72 edges, while nine additional subnetworks, each consisting of two to five genes, were also identified. Several nodes (e.g., Solyc02g088090.1.1, Solyc06g005170.3.1, and Solyc03g117980.3.1) displayed high connectivity with other genes.

To evaluate the biological significance of these interacting genes, gene ontology (GO) analysis was further performed. GO enrichment results within the biological process category corroborated signaling-centric functions: intracellular signal transduction (GO:0035556; *p* = 1.60 × 10^−14^), peptidyl–serine phosphorylation (GO:0018105; *p* = 2.45 × 10^−10^), protein autophosphorylation (GO:0046777; *p* = 2.45 × 10^−10^), and signal transduction (GO:0007165; *p* = 4.88 × 10^−12^) (Figure 5B). Molecular function enrichments included calcium ion binding (GO:0005509; *p* = 2.30 × 10^−21^), CaM-dependent protein kinase activity (GO:0004683; *p* = 3.91 × 10^−12^), and calcium-dependent protein serine/threonine kinase activity (GO:0009931; *p* = 3.91 × 10^−12^), underscoring Ca^2+^-centered regulation (Figure 5C).

Finally, highly connected hub genes with five or more interactions within the PPI networks were identified (Table 3). Of these, Solyc02g088090.1.1, encoding calmodulin (CALM), exhibited the highest connectivity with 13 genes, followed by Solyc06g005170.3.1, encoding mitogen-activated protein kinase. These hub genes showed strong representation within their respective modules, with module membership ranging from 0.68 to 0.95. Interestingly, with the exception of Solyc03g122340.3.1 encoding lipoxygenase D, six hub genes were involved in JCK1421-associated defense mechanisms, including the plant hormone signal transduction (map04075), plant–pathogen interaction (map04626), and MAPK signaling (map04016) pathways (Figure 4).

Taken together, the integrated PPI network analysis suggests that hub genes associated with *L. enzymogenes* JCK1421 treatment converge on a core set of regulators, highlighting a robust transcriptional architecture centered on signal perception, amplification, and downstream defense activation.

### 2.9. RT-qPCR Validation of RNA-Seq Data

RT-qPCR analysis of eight representative genes from each treatment comparison—control (C), JCK1421-treated (T), pathogen-inoculated (P), and co-treated (PT)—confirmed the RNA-seq expression trends (Figure S4). The direction and relative magnitude of changes were consistent across both methods, supporting the reliability of the transcriptomic data used for downstream analyses.

## 3. Discussion

Various enzymes and molecules produced by *L. enzymogenes* have been extensively investigated for their biological functions within the *Lysobacter* genus. The biosynthesis of lytic enzymes and HSAF by *L. enzymogenes* is a well-known antimicrobial strategy used for plant protection against pathogenic fungi, oomycetes, and even bacteria [8,9,11]. However, *L. enzymogenes* JCK1421 did not exhibit any antimicrobial activity against the pathogenic fungi or bacteria tested in this study.

The application of high-throughput sequencing technologies, such as transcriptome analysis, has revolutionized our understanding of complex biological interactions, enabling in-depth exploration of the dynamic interplay between pathogens and host plants [18]. Specifically, transcriptome analysis provides unbiased quantification of transcripts, offering comprehensive insights into key defense-related pathways and genes involved in plant–pathogen interactions [19,20]. *L. enzymogenes* JCK1421-treated tomatoes displayed a significant reduction in disease symptoms in response to *R. solanacearum* without directly inhibiting the pathogen. Based on these phenotypic assays, comparative transcriptome analysis with expression network and pathway enrichment was performed under conditions of *L. enzymogenes* JCK1421 treatment, with or without *R. solanacearum* infection, and identified JCK1421-associated modules. The salmon module from *L. enzymogenes* JCK1421 treatment alone and the red/yellow modules from combined JCK1421 and *R. solanacearum* treatments were extracted from the WGCNA modules. Functional network analysis of genes within these modules was linked to GO biological processes, including the MAPK cascade, regulation of the immune response, response to stress, and defense responses, providing specific mechanisms of JCK1421-mediated defense responses.

Although *R. solanacearum* infects via roots and spreads through xylem, foliar JCK1421 suppressed wilt without direct antibiosis, indicating induced systemic resistance (ISR). The transcriptomic data support a signaling-based mechanism involving MAPK cascades, Ca^2+^-dependent proteins, and JA/ET-related pathways that transmit defense cues from leaves to roots. This systemic priming likely strengthens root defenses and restricts pathogen colonization. Future studies on root transcriptomics will clarify these distal responses in more details.

Beneficial microorganisms such as plant growth-promoting rhizobacteria can reduce or eliminate plant biotic stresses by inducing systemic defense responses that confer broad-spectrum resistance to pathogens and insect herbivores. Beneficial microbes can induce mild but effective activation of plant immune responses in systemic tissues. Systemic resistance induced by different beneficials is regulated by jasmonate- and ethylene-dependent signaling pathways and is associated with priming for enhanced defense. In primed plants, defense responses are accelerated upon pathogen infection [21]. In this regard, *L. enzymogenes* JCK1421 represents a typical priming mechanism, based on transcriptome analyses and phenotypic assays. The key signaling pathway enriched in JCK1421-associated genes included a signal receptor (e.g., FLS2) or initiation components in the signaling pathway, independent of the pathogen. However, JCK1421-treated tomato plants, when challenged with the pathogen, activated diverse defense signaling pathways, including mid- and downstream components of signaling pathways. Thus, JCK1421 strongly triggers diverse signaling pathways involved in resistance responses against pathogen infections. For example, in jasmonate hormone signal transduction, genes (Solyc05g050280.3.1 and Solyc00g500216.1.1) encoding JAR1, which conjugates JA to isoleucine and functions directly in CORONATINE INSENSITIVE 1 (COI1)-mediated signal transduction [22], were enriched in JCK1421-treated genes as well as in JCK1421/pathogen co-treated genes. In contrast, genes (Solyc03g122190.3.1 and Solyc12g009220.2.1) encoding JAZ, which serve as co-receptors and transcriptional repressors of JA in plants [23], were enriched only in JCK1421/pathogen co-treated genes. Furthermore, the FLS2-mediated resistance response involves a cascade from the FLS2 receptor at the plasma membrane to WRKY transcription factors in the nucleus via MAPK signaling. FLS2 (Solyc02g070910.3.1) was enriched only among JCK1421-treated genes, whereas MAPK–WRKY genes were enriched only among JCK1421/pathogen co-treated genes.

In plants, Ca^2+^ signal transduction via calcium-conducting channels is an important mechanism for transmitting signals derived from diverse environmental stimuli [24,25]. CNGCs are components of Ca^2+^-conducting signal transduction pathways in response to hormones, including auxin, ethylene, and abscisic acid (ABA), as well as biotic and abiotic stresses [26]. Ca^2+^, an important secondary messenger in plant cells, changes rapidly in response to environmental cues. Specifically, Ca^2+^-binding proteins, including calmodulin/calmodulin-like (CaM/CML) and CDPK, are involved in essential early events during plant–pathogen interactions. Ca^2+^ influx is required in the early stages of immune activation and the hypersensitive response (HR), a type of programmed cell death occurring during avirulent pathogen infections [27,28]. Upon perception of conserved pathogen/microbial molecular patterns (PAMPs/MAMPs), a rapid increase in Ca^2+^ and NADPH oxidase-mediated reactive oxygen species (ROS) bursts is observed [28]. These responses lead to the activation of CDPKs and initiation of MAPK cascades to relay signals to the nucleus [29]. JCK1421-associated genes in response to pathogen infection were highly enriched in the CNGC–CDPK or CNGC–CaM/CML cascades, suggesting that Ca^2+^ influx is critical for JCK1421-mediated resistance to *R. solanacearum*. Furthermore, genes encoding calmodulin (Solyc02g088090.1.1 and Solyc03g098050.3.1) formed hubs with 13 and 7 connections, respectively, in the PPI network and were involved in plant–pathogen interaction, suggesting that calmodulin plays an important role in JCK1421-mediated resistance to *R. solanacearum.*

The MAPK pathways were also highly enriched, consistent with the enrichment of Ca^2+^-binding proteins. The MAPK pathway, a three-tiered kinase cascade (MAPKKK–MAPKK–MAPK), is involved in signaling multiple defense responses, including hormone biosynthesis, ROS generation, and HR [30,31]. JCK1421-mediated resistance to *R. solanacearum* enriched genes involved in a three-tiered kinase cascade, including MEKK1 (Solyc07g053170.4.1), MKK2 (Solyc12g009020.2.1), and MPK3 (Solyc06g005170.3.1). The gene encoding MPK3 was a central hub within the PPI network of genes in the salmon, red, and yellow modules associated with JCK1421 treatment. MPK3 was connected to 11 proteins involved in several GO biological processes, including the MAPK cascade, stress response, regulation of immune response, and defense response. MPK3 was directly connected to 1-aminocyclopropane-1-carboxylic acid synthase (ACS6), an enzyme that plays a key role in ethylene biosynthesis, and indirectly connected to WRKY22, which is involved in early defense responses to pathogens.

The PGPR-induced priming offers notable ecological advantages by enhancing the plant’s innate capacity to resist pathogens without continuous microbial presence or high metabolic costs. Such a state enables plants to respond more rapidly and effectively to subsequent infections while minimizing the need for chemical pesticides. This mechanism contributes to environmentally sustainable crop protection and supports the maintenance of beneficial microbial communities in the rhizosphere. This study mainly focused on transcriptomic and network-level analyses to reveal the signaling mechanisms underlying JCK1421-induced resistance. While these findings provide valuable insights into defense-related pathways, additional functional characterization of the key genes and signaling components will be important to further elucidate their specific roles and confirm the causal relationships underlying systemic resistance.

Overall, the mechanism of resistance induced by *L. enzymogenes* JCK1421 treatment in response to *R. solanacearum* involves a network of defense signaling, including activation of Ca^2+^-binding proteins such as CNGC and CDPK, as well as MAPK–transcription factor cascades, leading to hormone biosynthesis and early defense responses. These activated responses are very similar to those induced by other PGPR strains in response to abiotic stresses such as salt stress [32,33]. The CDPK and MAPK cascades represent central hubs of tolerance responses to salt stress. Thus, PGPR can activate common defense mechanisms against both biotic and abiotic challenges.

## 4. Materials and Methods

### 4.1. Bacterial and Fungal Strains

*Lysobacter enzymogenes* JCK1421 was isolated from pine forests located on Geumjeong Mountain (latitude 35.28015°, longitude 129.05062°) in Busan, South Korea. All bacterial strains used in this study, including *B. glumae*, *L. enzymogenes* JCK1421, *P. carotovorum*, and *P. syringae* DC3000, were cultured in Luria–Bertani (LB; Duchefa Biochemie, Haarlem, The Netherlands) medium. *R. solanacearum* was cultured in casamino acid peptone glucose (CPG; 1 g casamino acids/L, 10 g peptone/L, 5 g glucose/L) broth and incubated with shaking (200 rpm) at 28 °C. The optical density of bacterial cultures at 600 nm (OD600) was measured using a UV-1800 spectrophotometer (Shimadzu, Kyoto, Japan). All fungal strains, including *Fusarium oxysporum*, *Fusarium fujikuroi*, *Botrytis cinerea*, and *Colletotrichum scovillei*, were grown on yeast malt agar (YMA; MBcell, KisanBio., Seoul, Republic of Korea) and incubated at 28 °C. The list of bacterial and fungal strains used in this study, along with their hosts, associated diseases, optimal growth temperatures, and sources, is provided in Table S6.

### 4.2. In Vitro Interactions Between L. enzymogenes JCK1421 and Plant Pathogens

A spot-lawn assay [34] was modified and employed to test the antagonistic activity of *L. enzymogenes* JCK1421 against pathogenic bacteria using the appropriate agar medium for each species. Indicator lawns were prepared by inoculating plates with plant-pathogenic bacteria, and *L. enzymogenes* JCK1421 was spotted to assess growth inhibition. Control lawns were prepared without JCK1421 spotting. For fungal pathogens, a dual-culture assay [35] was modified by replacing PDA with YMA. Each fungal pathogen was inoculated at the center of the plate, while *L. enzymogenes* JCK1421 was streaked on either side (treatment) or omitted (control). Fungal growth inhibition was evaluated by measuring hyphal diameter.

### 4.3. Seedling Assay for Enhanced Disease Resistance by L. enzymogenes JCK1421

Tomato (*Lycopersicon esculentum* Mill. cv. Seokwang; Farhannong, Seoul, Korea) seeds were sown in square plug trays filled with horticultural nursery media (Punong, Republic of Korea) and grown in a growth chamber maintained at 25 °C under a 16 h light/8 h dark photoperiod. Seedlings were then transplanted into 7 cm × 6 cm pots containing the same potting soil. *L. enzymogenes* JCK1421 was cultured in LB broth for 24 h at 25 °C under constant agitation. The optical density of the culture was adjusted to OD_600_ = 0.8, and Tween-20 was added to a final concentration of 250 µg/mL. This suspension (20 mL) was foliar-sprayed onto tomato seedlings twice: 14 and 7 days prior to pathogen inoculation. For pathogen inoculation, *R. solanacearum* was cultured in CPG medium at 28 °C for 48 h, centrifuged at 4000× *g* for 5 min, and washed twice with distilled water. Three-week-old tomato plants were inoculated by applying a bacterial suspension adjusted according to optical density (OD_600_) readings and estimated soil weight per pot to achieve approximately 1 × 10^8^ CFU per gram of soil. Uninoculated and inoculated plants were cultivated in a growth chamber with a 14/10 h day/night cycle at 28 °C. Disease severity was evaluated 14 days after inoculation, when wilt symptoms are clearly distinguishable in tomato seedlings under controlled conditions, using a scale: 0, no wilting; 1, 1–20% wilting; 2, 21–40% wilting; 3, 41–60%; 4, 61–80% wilting; and 5, 81–100% wilting or plant death.

### 4.4. RNA Extraction from Tomato Seedlings

For transcriptome analysis, total RNA was extracted from tomato seedlings using a modified CTAB method [19]. Each RNA sample represented an independent biological replicate from a single plant, and three biological replicates per treatment were used for RNA sequencing. After surface sterilization with 70% ethanol and rinsing, 5 g of leaf tissue was ground in liquid nitrogen and homogenized in 15 mL of extraction buffer (100 mM Tris-HCl, 2% CTAB, 30 mM ethylenediaminetetraacetic acid (EDTA), 2 M NaCl, 0.05% spermidine, 2% polyvinylpolypyrrolidone, 2% 2-mercaptoethanol, and 1.5 mg/mL proteinase K). After incubation at 42 °C for 90 min and chloroform–isoamyl alcohol extraction, RNA was precipitated with 10 M LiCl, washed with ethanol, and resuspended in DEPC-treated water. RNA quality was confirmed by gel electrophoresis and quantified using a NanoDrop2000 spectrophotometer (Thermo Scientific, Barrington, IL, USA). Total RNA from treated tomato samples was also extracted using the RNeasy Mini Kit (Qiagen) according to the manufacturer’s instructions. RNA integrity and concentration were assessed with an Agilent 2100 Bioanalyzer (Agilent Technologies, Santa Clara, CA, USA). Ribosomal RNA was depleted using the NEBNext rRNA Depletion Kit (New England Biolabs, Ipswich, MA, USA).

### 4.5. RNA-Seq Library Preparation and Sequencing

For each sample, 1 µg of total RNA was used to construct complementary DNA (cDNA) libraries with the TruSeq RNA Sample Preparation Kit (Illumina, San Diego, CA, USA), following the manufacturer’s instructions, including mRNA enrichment via poly (A) selection. Libraries were indexed (unique dual indices), pooled equimolarly, and sequenced by Seeds (Daejeon, Republic of Korea) on an Illumina NovaSeq 6000 platform with 2 × 151 bp paired-end reads. Primary FASTQ quality was assessed using FastQC (https://www.bioinformatics.babraham.ac.uk/projects/fastqc/) (accessed on 15 March 2025) prior to downstream analyses. All sequencing data have been deposited in NCBI Gene Expression Omnibus under accession number GSE306380 (https://www.ncbi.nlm.nih.gov/geo/) (accessed on 23 March 2025).

### 4.6. Read Preprocessing, Genome Alignment, and Gene-Level Quantification

Raw FASTQ files were processed with the FASTX-Toolkit (https://github.com/agordon/fastx_toolkit/) (accessed on 13 April 2025) to remove residual primer/adapter sequences and low-quality reads. Primer/adapter clipping was performed using the *fastx_clipper* module, and reads shorter than 100 bp after trimming were discarded. Quality filtering was performed using the *fastq_quality_filter* module, requiring ≥50% of bases per read to have Phred ≥ 28 (Phred + 33 encoding, Illumina 1.8+). After trimming and filtering, an in-house Python (v3.8.10) script retained only properly paired reads and produced matched R1/R2 files for genome alignment. The *S. lycopersicum* reference genome SL4.0 and gene annotation ITAG4.0 were obtained from the Sol Genomics Network [36]. Preprocessed reads were aligned to the reference genome using BWA-MEM in paired-end mode with default seed length and scoring parameters [37]. SAM outputs were converted to BAM format, coordinate-sorted, and indexed using SAMtools [38]. Gene-level counts were generated with featureCounts against the ITAG4.0 annotation, assigning paired fragments to genes via exon features [39]. Read counts were normalized using the RPKM method for expression quantification.

### 4.7. WGCNA-Based Co-Expression Network Analysis

Expression values for 34,075 tomato genes were transformed to log_2_(RPKM + 1). Genes with zero expression across all 12 libraries were removed, resulting in a 24,367 × 12 expression matrix for co-expression network analysis. All analyses were conducted using the WGCNA package in the R statistical environment [40].

To identify potential outliers, libraries were clustered by average linkage based on Pearson correlation of the expression matrix. Candidate soft-thresholding powers (*β* = 1–20) were evaluated using the pickSoftThreshold module under a signed hybrid network type. A soft-thresholding power of *β* = 4 was selected, as this was the first value at which the scale-free topology fit exceeded R^2^ = 0.8 while preserving connectivity. Pairwise adjacency was computed from Pearson correlations, followed by calculation of the topological overlap matrix (TOM, type = signed). A dissimilarity measure (1 − TOM) was used for hierarchical clustering to generate a gene dendrogram. Modules were defined using dynamic tree cutting (deepSplit = 2, minClusterSize = 30), and module labels were assigned as colors.

Module eigengenes (MEs, the first principal component of each module) were computed and clustered to assess relationships among modules. Experimental traits were binary encoded according to treatment conditions (pathogen, JCK1421, and co-inoculation) for each library. Each ME was correlated with trait vectors using two-sided Pearson correlation. Modules of interest for biological interpretation were prespecified as those showing positive correlation with a given trait and *p*-value < 0.01. Gene significance (GS) was defined as |cor (expression, trait)|, and module membership (kME) as cor (expression, ME); GS–kME correlations were inspected to confirm intramodular coherence.

### 4.8. Pathway Enrichment Analysis

Protein-coding genes from the tomato genome were submitted to KAAS (KEGG Automatic Annotation Server) for KEGG orthology (KO) assignment [41]. The Eukaryotes reference set was used, and both single-directional best hit (SBH) and bi-directional best hit (BBH) strategies were applied. Hits were filtered using a score threshold of 60. KO assignments supported by BBH were preferentially retained; when BBH was absent, the top-scoring SBH was accepted if it met the same threshold. KO identifiers were mapped to pathway information from the KEGG pathway database (https://www.genome.jp/kegg/pathway.html) (accessed on 10 May 2025). For each module, pathway over-representation was tested using the hypergeometric distribution (*phyper*, upper-tail) in R. Four parameters were considered: (i) the total number of genes annotated to pathways in the tomato genome; (ii) the number of module genes included in ‘i’; (iii) the number of genes annotated to each pathway in the tomato genome; and (iv) the number of module genes annotated to each pathway. Pathways with *p*-value < 0.05 were considered significant. Molecular networks of enriched pathways were visualized using Cytoscape (v3.10.4) (https://cytoscape.org/) (accessed on 18 May 2025). Node/edge properties represented gene nodes, chemical nodes, and interaction types derived from KEGG pathway relationships; module membership was encoded as node color to facilitate interpretation of module-specific signals.

### 4.9. Protein–Protein Interaction Network and Hub Gene Analysis

Protein–protein interaction (PPI) networks were constructed using the STRING database v12.0 [42], with *S. lycopersicum* as the reference organism. A total of 106 genes from enriched pathways within JCK1421-associated co-expression modules were used as input. Interactions were retrieved with a medium confidence threshold (combined score ≥ 0.4), and unconnected nodes were excluded. The resulting networks were visualized using Cytoscape. Topological parameters, including degree, betweenness, and closeness centrality, were computed using the *NetworkAnalyzer* tool in Cytoscape. Hub genes were defined primarily by degree (≥5) and cross-validated by betweenness. Functional interpretation was supported by GO enrichment analysis, with significance defined as *p*-value < 0.05.

### 4.10. Validation of RNA-Seq Results by Reverse-Transcription Quantitative PCR (RT-qPCR)

To validate the RNA-Seq data, the expression of eight representative genes from each comparison group (C vs. T, C vs. P, and C vs. PT) was analyzed by RT-qPCR. The cDNA was synthesized from 1 µg total RNA with the Enzynomics cDNA Synthesis Kit (Enzynomics, Daejeon, Republic of Korea). Quantitative PCR was performed on a Rotor-Gene Q system (Qiagen, Hilden, Germany) with SYBR Green detection chemistry. Primer sequences are listed in Table S7. Thermal cycling conditions were 95 °C for 10 min, followed by 40 cycles of 95 °C for 10 s, 60 °C for 15 s, and 72 °C for 15 s. Each sample was run in triplicate. Relative transcript levels were calculated using the 2^−ΔΔCt^ method, normalizing to Actin-51 as the reference gene [43]. The resulting log_2_(fold change) values were directly compared with those obtained from RNA-Seq.

### 4.11. Statistical Analysis

Four treatments were established for the seedling assay: untreated control, tomatoes inoculated with *L. enzymogenes* JCK1421, tomatoes inoculated with *R. solanacearum*, and tomatoes co-inoculated with both *R. solanacearum* and *L. enzymogenes* JCK1421. Each treatment consisted of three independent experiments, with three biological replicates per experiment. Disease severity data were analyzed using one-way analysis of variance (ANOVA), followed by the least significant difference (LSD) test for pairwise comparisons (*p* < 0.05).

## 5. Conclusions

The present study highlights *L. enzymogenes* JCK1421 as a valuable model for investigating beneficial microbe-mediated priming of plant resistance and the activation of specific immune responses. Unlike other *L. enzymogenes* strains, JCK1421 does not display antimicrobial activity against pathogenic fungi or bacteria, yet it strongly induces resistance responses in tomato plants upon challenge with the pathogen *R. solanacearum*. Transcriptome and network analyses, combined with pathway enrichment, revealed that JCK1421-mediated resistance involves the induction of Ca^2+^-dependent proteins and channels—including calmodulin (CAM), calcium-dependent protein kinases (CDPKs), and cyclic nucleotide-gated channels (CNGCs)—along with the MAPK cascade. These signaling components converge on the activation of immune responses and hormone signaling pathways, particularly jasmonate and ethylene. Furthermore, RT-qPCR validation of selected genes confirmed the reliability of the RNA-Seq data, reinforcing the robustness of these transcriptomic findings. Collectively, these findings establish *L. enzymogenes* JCK1421 as a promising biological agent for crop protection, offering an environmentally friendly alternative to chemical pesticides in sustainable agriculture.

## Figures and Tables

**Table 1:** Summary of the *Solanum lycopersicum* (cv. Heinz 1706) reference genome assembly SL4.0 and annotation ITAG4.0 used in this study.

Feature	Value
Organism	*Solanum lycopersicum* (tomato)
Genome source	Sol Genomic Network (SGN) ^1^
Genome version	SL4.0
Annotation source	International Tomato Annotation Group (ITAG)
Annotation version	ITAG4.0
Total scaffold length (bp)	782,520,033
Number of scaffolds	13
Scaffold L50	6
Scaffold N50 (bp)	65,269,487
Total contig length (bp)	782,475,302
Number of contigs	448
Contig L50	37
Contig N50 (bp)	6,007,830
Total CDS length (bp)	34,993,605

^1^ Available at: https://solgenomics.net/ftp/tomato_genome/annotation/ITAG4.0_release/ (accessed on 13 December 2024).

**Table 2:** KEGG enrichment results in yellow and red modules.

Module	KEGG ID	KEGG Pathway	Count	*p*-Value
Yellow	map04626	Plant–pathogen interaction	12	6.56 × 10^−8^
	map04016	MAPK signaling pathway—plant	10	4.41 × 10^−6^
	map04912	GnRH signaling pathway	4	3.13 × 10^−5^
	map04075	Plant hormone signal transduction	9	0.0003
	map00592	alpha-Linolenic acid metabolism	4	0.0036
	map02010	ABC transporters	3	0.0041
	map00564	Glycerophospholipid metabolism	6	0.0046
	map04361	Axon regeneration	3	0.0076
	map00591	Linoleic acid metabolism	2	0.0087
	map04976	Bile secretion	2	0.0087
	map05231	Choline metabolism in cancer	3	0.0125
	map04540	Gap junction	2	0.0141
	map04910	Insulin signaling pathway	3	0.0358
	map00565	Ether lipid metabolism	2	0.0364
	map04024	cAMP signaling pathway	2	0.0457
	map05235	PD-L1 expression and PD-1 checkpoint pathway in cancer	2	0.0457
	map05418	Fluid shear stress and atherosclerosis	2	0.0457
Red	map04075	Plant hormone signal transduction	7	0.0001
	map04727	GABAergic synapse	3	0.0004
	map04626	Plant–pathogen interaction	5	0.0017
	map04814	Motor proteins	3	0.0028
	map05418	Fluid shear stress and atherosclerosis	2	0.0126
	map02024	Quorum sensing	2	0.0223
	map00040	Pentose and glucuronate interconversions	2	0.0342
	map04621	NOD-like receptor signaling pathway	2	0.0342

**Table 3:** Hub genes with the highest connectivity in the PPI network.

Gene ID	Degree	Module Membership	Symbols	Description	KEGG Pathway
Solyc02g088090.1.1	13	0.68	CALM	Calmodulin	Plant–pathogen interaction
Solyc06g005170.3.1	11	0.95	MPK3	Mitogen-activated protein kinase	MAPK signaling pathway
Solyc03g117980.3.1	9	0.93	RBOH	Respiratory burst oxidase	Plant–pathogen interaction
Solyc03g098050.3.1	7	0.86	CALM	Calmodulin	Plant–pathogen interaction
Solyc08g076930.1.1	7	0.74	MYC2	Transcription factor MYC2	Plant hormone signal transduction
Solyc03g122340.3.1	6	0.84		Lipoxygenase D	α-Linolenic acid metabolism
Solyc12g009020.2.1	5	0.85	MKK2	MAP kinase 2	MAPK signaling pathway

## Data Availability

The original RNA-seq data presented in this study are publicly available in the NCBI Gene Expression Omnibus (GEO) database (https://www.ncbi.nlm.nih.gov/geo/, 25 August 2025) under the accession number GSE306380.

## Supplementary Materials

The following supporting information can be downloaded from https://www.mdpi.com/article/10.3390/plants14223415/s1, Figure S1: In vitro interactions among *L. enzymogenes* JCK1421 and plant pathogens. (A) Dual-culture assay of JCK1421 against plant-pathogenic fungi on YMA medium. Each fungal pathogen was inoculated at the center of the plate, while JCK1421 was streaked on both sides (treatment), or omitted (control). Fungal growth inhibition was evaluated by measuring the hyphal diameter. Experiments were performed in triplicate. (B) Effect of JCK1421 on the hyphal growth of plant-pathogenic fungi in dual-culture assay. Hyphal diameters were measured in control plates (white bars) and in the presence of JCK1421 (gray bars). Data represent the mean ± standard deviation of three independent experiments. (C) Spot-lawn assay of JCK1421 against plant-pathogenic bacteria on LB or CPG medium. Indicator lawns were prepared by inoculation with plant-pathogenic bacteria, and JCK1421 was spotted to assess growth inhibition. Control lawns lacked JCK1421 spotting. Experiments were performed in triplicate. (D) Graph of inhibition zone diameters (mm) from spot-lawn assays conducted on LB medium. No inhibition was detected for any treatment (all values = 0, n.d.). Figure S2: KEGG pathway enrichment analysis of the salmon co-expression module. Significantly enriched pathways are visualized as a horizontal bar chart. The *x*-axis indicates the number of genes associated with each pathway, and the *y*-axis lists the corresponding KEGG pathways. Color intensity of each bar represents the statistical significance of enrichment (*p*-value), with darker colors denoting more significant enrichment. Figure S3: KEGG pathway enrichment analysis of the turquoise co-expression module. Significantly enriched pathways are visualized as a horizontal bar chart. The *x*-axis indicates the number of genes associated with each pathway, and the *y*-axis lists the corresponding KEGG pathways. Color intensity of each bar represents the statistical significance of enrichment (*p*-value), with darker colors denoting more significant enrichment. Figure S4: Validation of RNA-Seq expression profiles by RT-qPCR. Comparative analysis of eight representative genes selected from each treatment group using RT-qPCR and RNA-Seq. Expression values are shown as log_2_(fold change) relative to the control. (A) Control vs. JCK1421 (T); (B) Control vs. pathogen (P); (C) Control vs. JCK1421 + pathogen (PT). Each value represents the mean ± SE of three biological replicates. *Actin-51* was used as an internal reference gene. The consistent expression patterns between RT-qPCR and RNA-Seq confirm the reliability of the transcriptome data. Table S1: Summary of raw sequencing data. Table S2: Summary of read preprocessing statistics. Table S3: RPKM expression of tomato genes across 12 transcriptome datasets. Table S4: Network topology metrics for soft-thresholding power selection. Table S5: Gene distribution across WGCNA co-expression modules. Table S6: Pathogenic bacterial and fungal strains used in this study [35,44,45,46,47,48,49]. Table S7: Primer pairs of DEGs used for qRT-PCR validation.
