# Supplementary material for: Transcriptomic Analyses of Tomato Exhibiting Induced Resistance to Ralstonia solanacearum by Lysobacter enzymogenes JCK1421
Source: Plants (Basel). 2025 Nov 7;14(22):3415. doi: 10.3390/plants14223415 (PMC12655745; doi:10.3390/plants14223415)
Supplement: Supplementary file 1 [file plants-14-03415-s001.zip › 092225_Supplementary_figures - Oct28 - MM - R1.pdf]

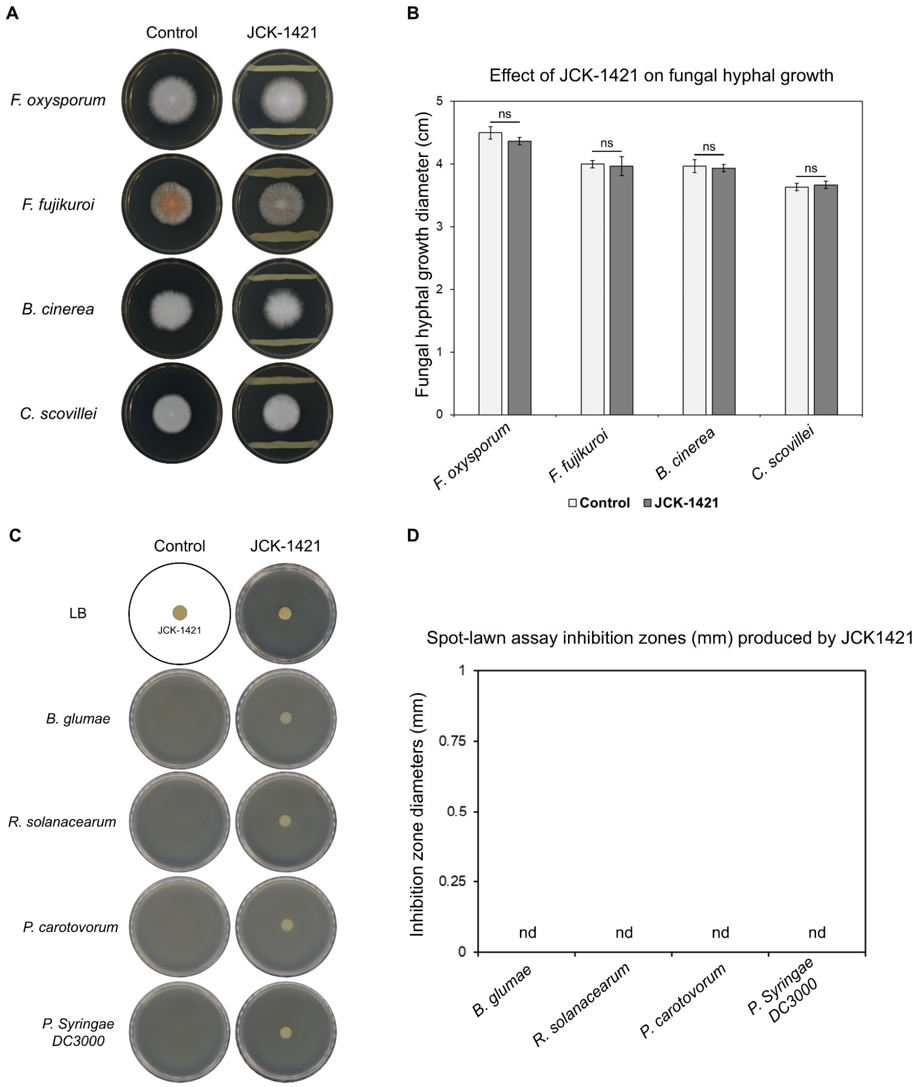

Figure S1. ***In vitro*** **interactions among** ***L. enzymogenes*** **JCK1421and plant pathogens.** (A) Dual culture assay of JCK-1421 against plant pathogenic fungi on YMA medium. Each fungal pathogen was inoculated at the center of the plate, while JCK-1421 was streaked on both sides (treatment), or omitted (control). Fungal growth inhibition was evaluated by measuring the hyphal diameter. Experiments were performed in triplicate. (B) Effect of JCK-1421 on the hyphal growth of plant pathogenic fungi in dual culture assay. Hyphal diameters were measured in control plates (white bars) and in the presence of JCK-1421 (gray bars). Data represent the mean ± standard deviation of three independent experiments. (C) Spot-lawn assay of JCK-1421 against plant pathogenic bacteria on LB or CPG medium. Indicator lawns were prepared by inoculation with plant pathogenic bacteria, and JCK-1421 was spotted to assess growth inhibition. Control lawns lacked JCK-1421 spotting. Experiments were performed in triplicate. (D) Graph of inhibition zone diameters (mm) from spot-lawn assays conducted on LB medium. No inhibition was detected for any treatment (all values = 0, n.d.).

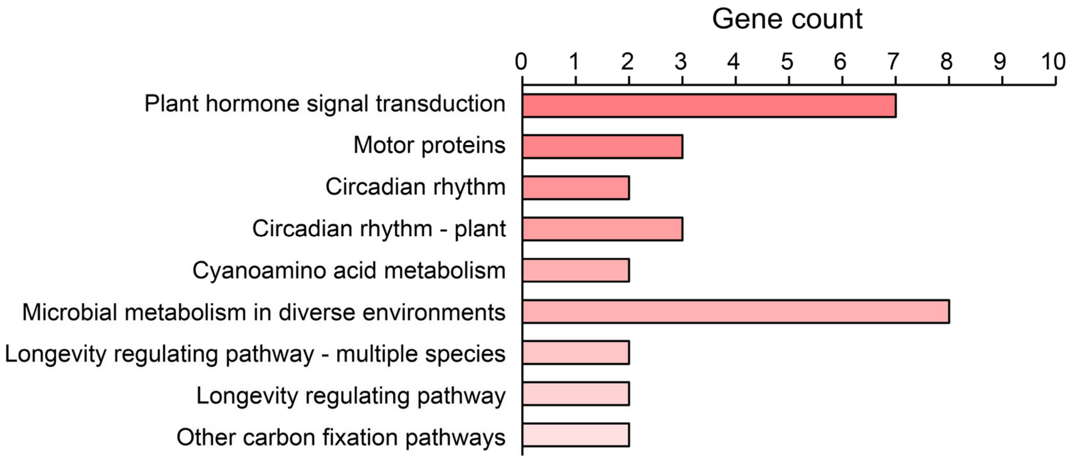

Figure S2. KEGG pathway enrichment analysis of the salmon co-expression module. Significantly enriched pathways are visualized as a horizontal bar chart. *x*-axis indicates the number of genes associated with each pathway, and the *y*-axis lists the corresponding KEGG pathways. Color intensity of each bar represents the statistical significance of enrichment (*p*-value), with darker colors denoting more significant enrichment.

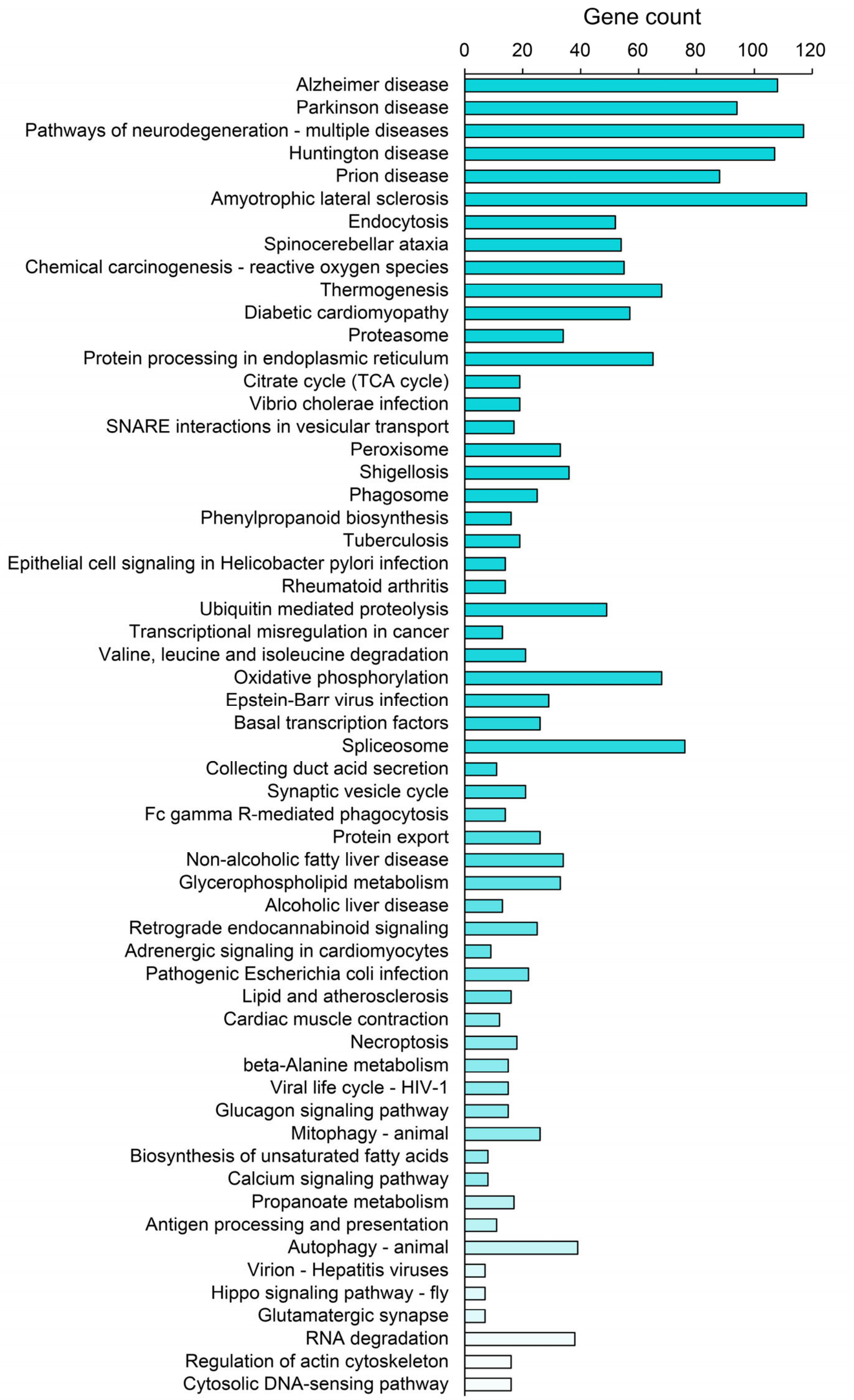

Figure S3. KEGG pathway enrichment analysis of the turquoise co-expression module. Significantly enriched pathways are visualized as a horizontal bar chart. *x*-axis indicates the number of genes associated with each pathway, and the *y*-axis lists the corresponding KEGG pathways. Color intensity of each bar represents the statistical significance of enrichment (*p*-value), with darker colors denoting more significant enrichment.

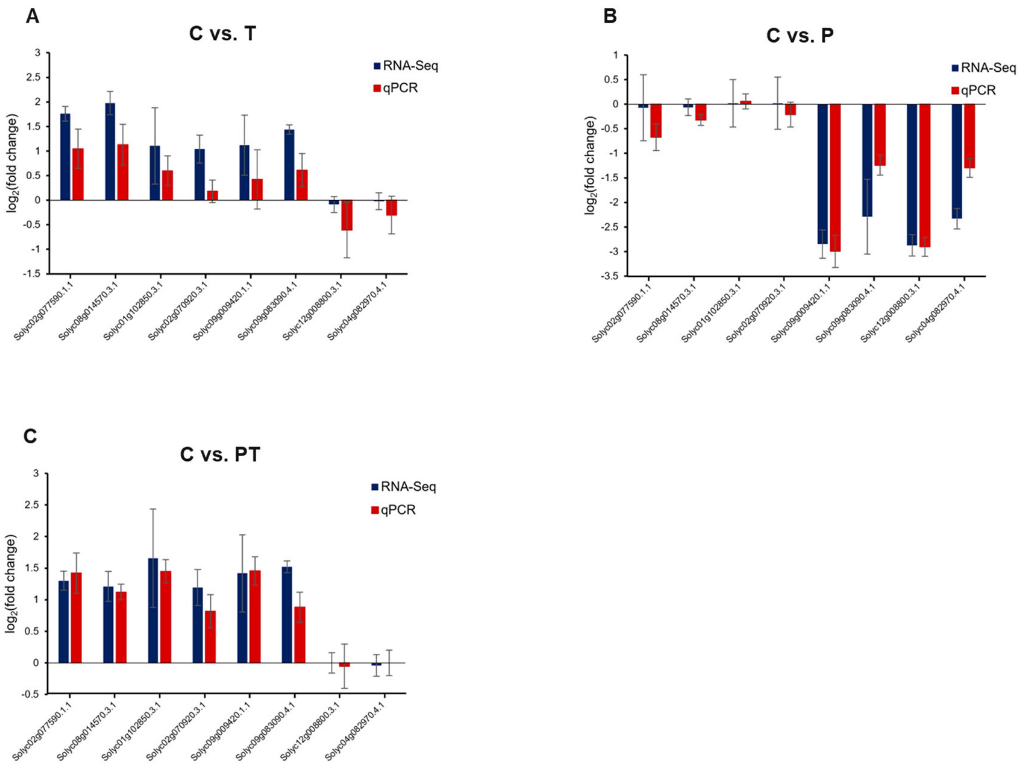

Figure S4. Validation of RNA-Seq expression profiles by RT-qPCR. Comparative analysis of eight representative genes selected from each treatment group using RT-qPCR (red bars) and RNA-Seq (blue bars). Expression values are shown as $\log_2$(fold change) relative to the control. (A) Control vs. JCK1421 (T); (B) Control vs. Pathogen (P); (C) Control vs. JCK1421 + Pathogen (PT). Each value represents the mean ± SE of three biological replicates. *Actin-51* was used as an internal reference gene. The consistent expression patterns between RT-qPCR and RNA-Seq confirm the reliability of the transcriptome data.
